# The renoprotective effects of Heme Oxygenase-1 during contrast-induced acute kidney injury in preclinical diabetic models

**DOI:** 10.6061/clinics/2021/e3002

**Published:** 2021-10-05

**Authors:** Cassiane Dezoti da Fonseca, Mirian Watanabe, Sheila Marques Fernandes Couto, Alef Aragão Carneiro dos Santos, Fernanda Teixeira Borges, Maria de Fatima Fernandes Vattimo

**Affiliations:** I Escola Paulista de Enfermagem, Universidade Federal de Sao Paulo, Sao Paulo, SP, BR.; II Centro Universitario das Faculdades Metropolitanas Unidas (FMU), Sao Paulo, SP, BR.; III Laboratorio Experimental de Modelos Animais (LEMA), Escola de Enfermagem, Universidade de Sao Paulo, Sao Paulo, SP, BR.; IV Programa de Pos-graduacao Interdisciplinar em Ciencias da Saude, Universidade Cruzeiro do Sul, Sao Paulo SP, BR.; V Divisao de Nefrologia, Universidade Federal de Sao Paulo, Sao Paulo, SP, BR.

**Keywords:** Heme Oxygenase-1, Nephrotoxicity, Oxidative Stress, Diabetes Mellitus, Iodinated Contrast

## Abstract

**OBJECTIVES:**

Contrast-induced acute kidney injury (CI-AKI) is an important clinical problem that can be aggravated by diabetes mellitus, a major risk factor. However, heme oxygenase-1 (HO-1), a promising therapeutic target, can exert antioxidant effects against CI-AKI. Thus, we investigated the role of HO-1 in CI-AKI in the presence of diabetes mellitus.

**METHODS:**

Twenty-eight male Wistar rats weighing 250-300g were subjected to left uninephrectomy, and concomitantly, diabetes induced by streptozotocin (65 mg/kg). After 12 weeks, iodinated contrast (meglumine ioxithalamate, 6 mL/kg) and hemin (HO-1 inducer-10 mg/k) were administered 60 min before iodinated contrast treatment. The rats were randomly divided into four groups: control, diabetes mellitus (DM), DM iodinated contrast (DMIC), and DMIC hemin (DMICH). Kidney function, albuminuria, oxidative profile, and histology were assessed. All experimental data were subjected to statistical analyses.

**RESULTS:**

CI-AKI in preclinical diabetic models decreased creatinine clearance and increased urinary neutrophil gelatinase-associated lipocalin (NGAL) levels and the degree of albuminuria. Additionally, the levels of oxidative and nitrosative stress metabolites (urinary peroxides, thiobarbituric acid-reactive substances, and NO) were elevated, while thiol levels in kidney tissue were reduced. Kidney histology showed tubular cell vacuolization and edema. HO-1 inducer treatment improved kidney function and reduced urinary the NGAL levels. The oxidative profile showed an increase in the endogenous thiol-based antioxidant levels. Additionally, the tubular injury score was reduced following HO-1 treatment.

**CONCLUSIONS:**

Our findings highlight the renoprotective effects of HO-1 in CI-AKI and preclinical diabetic models. Therefore, HO-1 ameliorates kidney dysfunction, reduces oxidative stress, and prevents cell necrosis.

## INTRODUCTION

Contrast-induced acute kidney injury (CI-AKI) is an iatrogenic syndrome that arises after iodinated contrast (IC) administration, which is used to perform diagnostic imaging tests such as cardiac catheterization (1,2). Its incidence varies from 3% to 19%, but risk factors such as chronic kidney disease, advanced age, and diabetes mellitus (DM) can increase it to 26% (3-5). In this context, DM is associated with microvascular comorbidities such as nephropathy, a chronic condition that exacerbates kidney vulnerability to toxic agents such as IC (6-8).

CI-AKI is clinically characterized as kidney dysfunction, with elevated serum creatinine greater than or equal to 0.5 mg/dL or less than 25% of baseline between 48 and 72 hours after IC injection, associated with anuria, or oliguria and electrolyte imbalance (5,9). Chronic hyperglycemia can intensify hypoxic, nitrosative, and oxidative stress following IC administration (4,8,10).

The pathophysiology of CI-AKI is poorly understood. Hypoxia is the main mechanism underlying CI-AKI and is caused by prolonged vasoconstriction that induces adhesion molecule expression, leukocyte-endothelium interaction, and activation of inflammatory mediators such as cytokines and eicosanoids (2,8-10). Nevertheless, high osmolality and viscosity of IC may enhance its toxic effect in the kidney tubules by increasing intratubular pressure and reducing kidney blood flow (KBF) and glomerular filtration rate (GFR). *In vivo* and *in vitro* studies corroborate this hypothesis (5,9,11). Additionally, direct tubular toxicity caused by IC can stimulate the production of reactive oxygen species (ROS), which affect mitochondrial and nuclear DNA, lipid membranes, and cellular proteins, leading to cell apoptosis and necrosis (12-16).

Heme oxygenase 1 (HO-1) is an inducible enzyme with potent antioxidant, anti-inflammatory, and anti-apoptotic properties. HO-1 activity occurs via heme breakdown and generation of protective products such as carbon monoxide (CO) and biliverdin, with the subsequent formation of bilirubin and ferritin via iron release from the heme moiety (17). Numerous studies conducted with animals have highlighted the promising effects of HO-1 induction, showing its antioxidant and anti-inflammatory properties in kidney diseases (18-20).

The explosion of therapeutic and diagnostic interventions, especially cardiac procedures that require IC, can contribute to the unfavorable epidemiology of CI-AKI, as well as complications in diabetic patients. We hypothesized that chronic hyperglycemia, which confers a predisposition to the occurrence of CI-AKI and HO-1 induction via hemin, could protect the kidney due to vasodilation, anti-inflammatory, antioxidant, and antiapoptotic effects. In this study, we aimed to investigate the protective effects of HO-1 on CI-AKI in diabetic rats by analyzing metabolic functions, oxidative profile, and histopathological staining.

## MATERIALS AND METHODS

Ethics: All procedures in this study were approved by the Committee on Animal Research and Ethics of the University of São Paulo (Protocol 060/128/022012) and were conducted in accordance with international standards for the care and use of laboratory animals.

Animals: Twenty-eight adult male Wistar rats weighing 280-390 g were used in this study. They were housed in a temperature-controlled room (25°C/77°F), with 12-hour light-dark cycles and had free access to water and rat chow (Nuvilab CR-1, Nuvital, Brazil).

### Procedures and timing

The animals were randomly divided into four groups: (1) citrate-control group (C): left unilateral nephrectomy was performed on day 1, and on day 20, citrate buffer was administrated (0.01 M (pH 4.2); in the caudal vein, intravenous - i.v.), being housed under controlled conditions during 12 weeks; (2) DM: DM was induced by streptozotocin (STZ) administration (65 mg/kg, diluted in 0.01 M citrate buffer (pH 4.2), i.v.) on day 20, and the animals stayed under controlled conditions during 12 weeks (21); (3) DM + IC (DMIC): DM animals received 6 mL/kg meglumine ioxithalamate, i.p. on day 85 (10); (4) DM + IC + H (DMICH): DM animals received IC and hemin (HO-1 inducer, 10 mg/kg i.p. 60 minutes before IC) on day 85 (22).

The blood glucose levels of the animals used to establish the diabetic model were assessed 48h after the induction of diabetes, using Advantage urine reagent strips (Advantage, Roche^®^, Brazil). The animals with blood glucose levels above 250 mg/dL were considered diabetic. All diabetic animals had their body weight assessed, and their blood glucose was monitored once a week for 12 weeks (85 days). Metabolic cages were used for the urine sample collection. On day 85, immediately after the last injection, the rats were placed in metabolic cages for a 24-hour urinary volume measurement and urine sample collection.

### Blood sample collection

The animals were anesthetized with a ketamine/xylazine mixture (75 mg/kg/10 mg/kg; Anasedan^®^, Vetbrands) via intraperitoneal injection to collect blood samples through abdominal aorta puncture. During the procedure, they were kept on a surface heated to 38°C to avoid hypothermia. Euthanasia was performed by exsanguination, a physical method, according to the ethical standards for animal use in research laboratories (23).

### Tissue sample collection/preparation

The left and right kidneys were prepared for the measurement of the levels of antioxidant enzymes and histological analysis.

### Kidney function

According to well-established methods (24), serum and creatinine concentrations in the urine were measured using the Jaffe method, and creatinine clearance was calculated based on the following formula: creatinine clearance=[urine creatinine concentration (mg/dL)×24-hour urinary volume (mL/min)]/serum creatinine concentration (mg/dL). The calculated creatinine clearance rate (mL/min) was normalized to the weight of the rats (25).

### Urinary neutrophil gelatinase-associated lipocalin (NGAL)

Urine NGAL levels were analyzed using the Rat-NGAL ELISA kit, BioVendor, research, and diagnostic products (11).

### Urinary albumin

Albumin concentrations in 24-hour urine samples were assessed using commercially available enzyme-linked immunosorbent assays (ELISA) (Bethyl Laboratories, USA). The optical density of each sample was determined using an Ultra Microplate Reader (EL808; Bio-Tek Instruments, Winooski, VT, USA) and expressed as ng/24 h for urine concentrations (26).

### Oxidative profile

As described previously (24), (i) urinary peroxides were determined based on version 2 of the ferrous oxidation-xylenol orange (FOX-2) method. Xylenol orange shows a high selectivity for Fe^3+^, producing a blue/purple complex (A=4.3×10^4^ M^-1^ cm^-1^). Values were corrected for urine creatinine content (in grams) and were expressed as nmol/g creatinine (27). (ii) Non-protein soluble thiols in the kidney were assessed by tissue homogenization in 1 mL of a solution containing 10 mM sodium acetate, 0.5% Tween 20, and 100 µM DTPA (pH 6.5). The first aliquot was reserved for the immediate measurement of total protein content. The second aliquot was precipitated with 20% trichloroacetic acid (1:1) to measure the total thiol content. Deproteinized samples were homogenized in 100 µL of a solution containing 100 mM Tris buffer (pH 8.0). After 10 min at room temperature, the quantity of thiols was determined based on the mean absorbance at 412 nm (A=13.6×10^3^ M^-1^ cm^-1^). The concentration of soluble thiols was corrected for the total protein content and expressed as nmol/mg total protein (28). (iii) Lipid peroxidation levels of malondialdehyde (MDA) were determined by measuring the levels of thiobarbituric acid-reactive substances (TBARS). For peroxidation quantification, 0.4 mL of a urine sample mixed with 0.6 mL water was added to a reaction mixture consisting of 1.0 mL 17.5% trichloroacetic acid (TCA) and 1.0 mL 0.6% thiobarbituric acid. This mixture was heated in a water bath at 95°C for 20 min, removed, cooled on ice, and subsequently mixed with 1.0 mL 70% TCA. The solution was homogenized and incubated for 20 min. Finally, the solution was read in a spectrophotometer at 535 nm (A=1.56×10^5^ M^-1^ cm^-1^. Values are expressed as nmol/g creatinine (29). (iv). Nitric oxide (NO) was determined in urine samples using the Griess method. Briefly, a mixture of 1% sulfanilamide in 5% H_3_PO_4_ and 0.1% naphthylethylenediamine solution (Sigma-Aldrich, Saint Louis, USA) was added to the samples, which were read in a spectrophotometer at 546 nm. The levels of nitrite, a stable NO metabolite, was estimated from a standard curve constructed using NaNO_2_. Creatinine level assessment in the urine was performed as previously described and was used to normalize the results (30,31).

### Histological analysis

According to well-established methods (24), slices of the left kidney were immersed in Bouin’s solution for 4 h. Subsequently, kidney tissues were placed in a series of baths of 70% alcohol for picric acid elimination, dehydrated, and embedded in paraffin. Paraffin sections of perfused/fixed kidneys were stained with hematoxylin and eosin for histological analysis via light microscopy. Tubulointerstitial changes were defined as the presence of epithelial cell edema, vacuolar degeneration, necrosis, and desquamation of tubular cells. Tubular injury quantification was scored as follows: 0=less than 5% of the focal areas; 1=involvement of less than 5-25% of the kidney cortex; 2=involvement of less than 25-50% of the kidney cortex; 3=involvement of 50-75% of the kidney cortex; and 4=involvement of more than 75% of the kidney cortex. The slides were read using an optical microscope Axioskop 40 (Carl Zeiss, Jena, Germany) (32,33).

### Statistical analysis

All quantitative data are expressed as the mean±SEM. Analysis of variance (ANOVA) was performed along with confidence intervals around the means and pairwise comparisons using the Newman-Keuls post hoc test. Non-continuous variables were available in non-parametric Kruskal-Wallis, following the Dunn test. All statistical analyses were performed using the Graph-Pad Prism version-7 for Windows^®^. Statistical significance was defined as *p*<0.05.

## RESULTS

### The effects of HO-1 on physiological parameters and kidney function

As shown in Table 1, physiological parameters confirm the establishment of the diabetic model, which presents hyperglycemia, polydipsia, polyphagia, and polyuria, indicated by increased food intake, water intake, blood glucose, and urine output elevation, respectively. The animals in the DM group presented with kidney hypertrophy and increased urinary albumin (*p*<0.001), as demonstrated by the elevation of kidney/animal weight ratio and urinary volume. Interestingly, the highest ratio was observed in the animals that received IC. Furthermore, urinary albumin levels increased in the animals from the DM group. Treatment with hemin prevented the decrease in GFR, demonstrating the renoprotective action of HO-1 in the DMICH group, compared to the case for the control DMIC group. Urinary NGAL levels were lower in the DMICH group than in the DMIC group.

### The effects of HO-1 on oxidative stress

**Figure 1:**
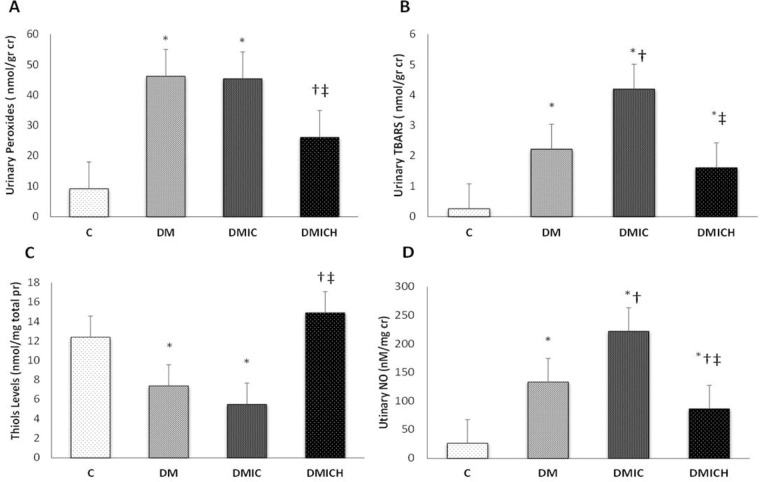
Oxidative and nitrosative profiles of the rats from the control and diabetic groups. **(A)** Urinary peroxide levels (nmol/g cr), **(B)** urinary TBARS levels (nmol/g cr), **(C)** thiol levels (nmol/mg total pr), and **(D)** urinary NO levels (nM/mg cr). Cr: creatinine. Pr: protein. NO: nitric oxide. Data are shown as the mean±SEM for each group (n=7). Statistical significance was set at *p*<0.05. *p<0.05 *versus* C, †*p*<0.05 *versus* DM, ‡*p*<0.05 *versus* DMIC.

The levels of urinary peroxides (UP, Figure 1A) and urinary TBARS (Figure 1B) in the DM and DMIC groups showed a significant increase (UP: DM: 46.2±21.8 nmol/g cr, DMIC: 45.4±10.2 nmol/g cr *versus* C: 9.2±2.7 nmol/g cr; TBARS: DM: 2.22±0.61 nmol/g cr; DMIC: 4.20±1.04 nmol/g cr *versus* C: 0.26±0.07 nmol/g cr, *p*<0.05). Treatment with hemin reduced the production of oxidative metabolites (UP: DMICH: 26.1±13.1 nmol/g cr *versus* DM: 46.2±21.8 nmol/g cr and DMIC: 45.4±10.2 nmol/g cr; TBARS: DMICH: 1.61±0.26 nmol/g cr *versus* DM: 2.22±0.61 nmol/g cr and DMIC: 4.20±1.04 nmol/g cr, *p*<0.05).

On the other hand, the thiol levels (Figure 1C) showed a reduction in the DM and DMIC groups (DM: 7.4±2.9 nmol/mg pr, DMIC: 5.5±1.9 nmol/mg pr *versus* C: 12.4±4.6, *p*<0.05). Nevertheless, when the HO-1 inducer was administered, the thiol levels were restored (DMICH: 14.9±3.7 nmol/mg pr *versus* DM: 7.4±2.9 nmol/mg pr, DMIC: 5.5±1.9 nmol/mg pr, *p*<0.05).

Urinary NO (Figure 1D) is associated with nitrosative stress in the urinary system, mainly in the kidneys. The elevation of this parameter was observed in the DM and DMIC groups (DM: 133.5±23.7 nM/mg cr U, DMIC: 222.2±33.8 nM/mg cr U *versus* C: 26.5±10.9 nM/mg cr U, *p*<0.05). Treatment with hemin significantly reversed the increase in nitrate excretion, resulting in the stabilization of NO metabolism (DMICH: 86.5±17.4 nM/mg cr U *versus* DM: 133.5±23.7 nM/mg cr U; DMIC: 222.2±33.8 nM/mg cr U, *p*<0.05).

### The effects of HO-1 treatment on kidney tissues

**Figure 2:**
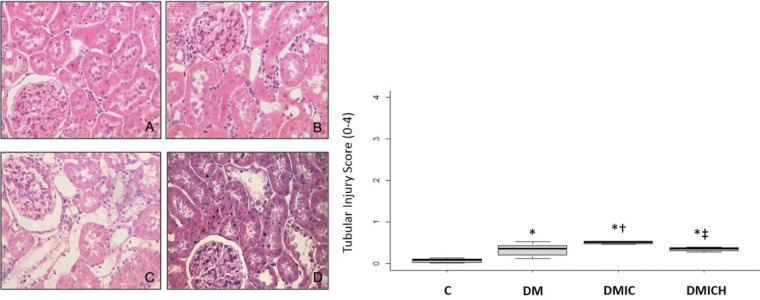
Mean (±SEM) of histological images and tubular injury score from the control and diabetic groups. Representative images of hematoxylin and eosin staining at 400× magnification. n=5-6 per each group. C (A), DM (B), DMIC (C), and DMICH (D). *p<0.05 *versus* C, †*p*<0.05 *versus* DM, ‡*p*<0.05 *versus* DMIC.

Figure 2 shows the histological parameters of the kidneys. Tubulointerstitial lesions accounted for less than 5% of the focal areas. Tubular vacuolar degeneration, edema, cell casts, and hyaline casts were observed. There was an increased infiltration of polymorphonuclear cells (Figure 2C), and the tubular injury score (0-4) was DMIC: 0.50±0.03 *versus* C: 0.08±0.10, DM: 0.33±0.15, *p*<0.05. Treatment with HO-1 restored the kidney histologic alterations and reduced the tubular injury score (DMICH: 0.34±0.05 *versus* DMIC: 0.50±0.03, *p*<0.05).

## DISCUSSION

CI-AKI is a serious iatrogenic complication in clinical practice. Cardiac hemodynamic procedures are responsible for a large proportion of these adverse events. DM is considered a major risk factor for CI-AKI. Hyperglycemia has been linked to high CI-AKI prevalence, demonstrating increased short- and long-term mortality. In this context, several agents are being investigated because of their possible renoprotective action against DM-associated CI-AKI, especially in preclinical and clinical models (5,10,34-38).

This study demonstrates, for the first time, that HO-1 can be an innovative strategy to prevent and treat CI-AKI by mitigating the increase in the levels of kidney injury biomarkers and improving kidney function, ameliorating oxidative stress, and normalizing the histological parameters in a preclinical diabetic risk factor model.

The exact pathogenesis of CI-AKI is not completely understood. Kidney vasoconstriction and direct nephrotoxicity are associated with kidney parenchymal hypoxia, generation of reactive oxygen species, and inflammatory processes, all of which play pivotal roles in kidney damage. In addition, the predisposition of diabetic kidneys to CI-AKI may be related to chronic hyperglycemia due to increased levels of NADPH oxidase and mitochondrial protein glycation in favor of oxidative stress, resulting in a state of glucose toxicity (2,6,8,10).

In the current study, we observed a significant increase in the levels of kidney injury biomarkers, creatinine clearance, urinary NGAL, and urinary albumin in diabetic rats that received contrast media. A previous study demonstrated that contrast media reduced serum creatinine and BUN levels in diabetic rats (39). Han et al. showed an increase of at least 25% in serum creatinine levels in diabetic patients after IC exposure (40). Fernandes et al. demonstrated a significant decrease in GFR (assessed by a gold standard method, inulin clearance) and increased levels of urinary NGAL in preclinical CI-AKI models in association with diabetic and chronic kidney disease (11). Albuminuria is closely related to glomerular changes in DM, such as basal glomerular thickening. Ptilovanciv et al. demonstrated the elevation of urinary albumin levels in diabetic rats (26), corroborating our data. HO-1 induction in this study prevented a decrease in GFR and restored the levels of kidney biomarkers. NGAL is a stress protein that is upregulated in residual viable kidney cells and is highly sensitive for the early detection of AKI (41,42). In kidney injuries, overexpression of NGAL could be related to an intensive oxidative stress mechanism. Some studies have demonstrated that HO-1 activity decreases NGAL levels (43,44).

Additionally, this study demonstrated an important increase in the levels of urinary peroxides, TBARS, and NO and a decrease in thiol levels by glutathione peroxidase consumption, resulting in oxidative stress in diabetic rats with CI-AKI. There is a synergic adverse event associated with IC administration and diabetes in the kidney in favor of oxidative stress. The mechanism underlying ROS formation can be related to deregulated kidney hemodynamics and endothelial dysfunction. Oxygen consumption affects mitochondrial and nuclear DNA, lipid membranes, and cellular proteins with accumulation of hypoxia-inducible factors (HIFs). Renal medullary hypoxic injury is characterized by apoptosis and necrosis. Moreover, metal cations (such as Fe^+2^ and Cu^+1^) in various redox-sensitive compounds, disconnected from their protein envelopes, may trigger the generation of ROS and catalyze the formation of highly toxic hydroxyl radicals from hydrogen peroxide and superoxide (9,12).

Endothelial nitric oxide synthase (eNOS) overproduction can combine to form peroxynitrite in association with superoxide by uncoupling tetrahydrobiopterin or arginine, which perpetuates oxidative stress (45). Pereira et al. demonstrated an important increase in TBARS levels in rats subjected to contrast agent administration (46). Hou et al. also showed a significant increase in the levels of oxidative stress markers, such as superoxide dismutase (SOD), MDA, and glutathione peroxidase (GSHPX) (39). In agreement with previous studies, it has been demonstrated that HO-1 induction has an antioxidant effect. Thus, this work is the first to demonstrate the effects of HO-1 in CI-AKI in a DM experimental model. HO-1 activity contributes to the scavenging of free radicals, such as peroxynitrite, by the overexpression of bile pigments, ultimately inhibiting lipid peroxidation. Additionally, the cyclic interconversion of biliverdin to bilirubin mitigates oxidative stress through the sequestration of hydrogen peroxide during the reaction (17,46). Fonseca et al. demonstrated that HO-1 induction increased the catalase and thiol levels and reduced the levels of oxidative metabolites in rats subjected to polymyxin B-induced nephrotoxicity (47). Studies with CI-AKI models suggest that several agents with antioxidant properties grant protection against oxidative stress through the modulation of HO-1 expression and activity (48,49).

From a histological perspective, the samples showed tubular epithelial vacuolization, luminal congestion, edema, and epithelial cell flattening, with the formation of intratubular casts and the presence of interstitial infiltrate in kidney cell structure in approximately 5% of kidney tubular cells. A recent clinical study demonstrated an increase in the serum creatine and cystatin C levels in diabetic patients exposed to contrast media (37). Similar to HO-1, an insulin-sensitizing hormone, AdipoRon, restored the kidney morphological parameters in a preclinical model of nephropathy established by the administration of the contrast agent iopromide (50).

From a translational perspective, DM could be a predictor of CI-AKI due to the strong association between contrast media imaging techniques and the presence of vascular disease, such as that observed in nephropathy. It should be noted that the incidence of CI-AKI is greater (almost 50%) in diabetic patients than in normoglycemic patients. Therefore, treatment with antioxidant substances and application of a risk prediction score need to be considered in all diabetic patients exposed to contrast media (1-3,5).

## CONCLUSION

In conclusion, this study showed that HO-1 inducer administration can reverse the decrease in GFR and prevent oxidative stress in diabetic rats after IC exposure. Thus, owing to its antioxidant effects, HO-1 represents a therapeutic alternative for treating patients at a risk of CI-AKI. Further experiments and randomized clinical trials are required to determine its protective role.

## AUTHOR CONTRIBUTIONS

Fonseca CD, Watanabe M, Couto SMF and Vattimo MFF conceived and designed the experiments. Fonseca CD, Watanage M, Couto SMF and Santos AAC performed the experiments. Fonseca CD and Couto SMF analyzed the data. Fonseca CD, Borges FT, Couto SMF and Santos AAC contributed reagents/materials/analysis tools. Fonseca CD, Borges FT and Vattimo MFF wrote the manuscript.

## Figures and Tables

**Table 1:** Physiological parameters and kidney function.

Variable	C	DM	DMIC	DMICH
Water intake (mL/24 h)	24±6	113±35*	198±39*†	169±21*†
Food intake (g/24 h)	13±5	25±8*	36±8*†	31±3*
Body glucose (mg/dL)	110±12	549±32*	567±48*	588±43*
KW/BW (×10^-3^)	0.52±0.13	0.78±0.07*	0.91±0.08*†	1.12±0.26*†
Urinary output (mL/min)	0.01±0.02	0.06±0.01*	0.09±0.01*†	0.10±0.01*†
Urinary creatinine (mg/dL)	69.2±7.6	12.2±2.7*	6.8±2.0*†	5.8±1.0*†
Serum creatinine (mg/dL)	0.29±0.04	0.64±0.05*	0.87±0.16*†	0.63±0.16*‡
Creatinine clearance (ml/min/100 g)	0.51±0.08	0.32±0.05*	0.19±0.03*†	0.40±0.07*‡
Urinary NGAL (pg/mL)	130.9±50.3	141.8±42.7	327.2±61.1*†	152.4±57.8‡
Urinary albumin (ng/24 h)	0.54±0.30	3.02±0.95*	4.03±0.87*	3.29±0.40*

KW, kidney weight; BW, body weight *n*=7/group. Data are shown as the mean±SEM for each group. Statistical significance was set at *p*<0.05. *p<0.05 *versus* C, †*p*<0.05 *versus* DM, ‡*p*<0.05 *versus* DMIC.
